# Cadmium contamination of soil and crops is affected by intercropping and rotation systems in the lower reaches of the Minjiang River in south-western China

**DOI:** 10.1007/s10653-015-9762-4

**Published:** 2015-09-01

**Authors:** Yang Liu, Kai Liu, Yong Li, Wanqin Yang, Fuzhong Wu, Peng Zhu, Jian Zhang, Lianghua Chen, Shun Gao, Li Zhang

**Affiliations:** Key Laboratory of Ecological Forestry Engineering in Sichuan Province, Institute of Ecology & Forestry, Sichuan Agricultural University, Chengdu, 611130 China; Sichuan Forestry Exploration and Design Institute, Chengdu, 610081 China; Collaborative Innovation Center of Ecological Security in the Upper Reaches of Yangtze River, Chengdu, China

**Keywords:** Cadmium, Intercropping, Rotation, Arable soil, Food safety

## Abstract

Cadmium (Cd) accumulation and pollution in arable soils are particularly serious in the lower reaches of the Minjiang River in southwest of China. In this study, the remediation efficiency of Cd contamination in arable soils, the distribution pattern of Cd concentration in crops, and the food safety to humans of three typical cropping systems (S1: maize + sweet potato—Chinese cabbage, S2: maize + ginger—stem mustard, and S3: rice) were investigated and evaluated. After 1-year rotation, the percentage of Cd extracted by crops from the plough soil layer was observed in three system fields with the trend of S1 (2.30 %) > S2 (1.16 %) > S3 (0.21 %) and Cd extraction amount in crops was maximum in sweet potato, then in maize. The same kind of crop had the same pattern of Cd distribution in organs, and the edible parts generally accumulated less Cd amount than the inedible parts. Further, the grain crops were found to possibly be suitable one for using as phytoaccumulators of Cd contamination for farmlands. Direct consumption of these crops from the three systems would pose a high health risk to local inhabitants since it would result in the monthly intake of Cd (247 μg kg^−1^ body weight) being nearly 10 times higher than the recommended tolerable monthly intake (RTMI) (25 μg kg^−1^ body weight), resulting mainly from the consumption of vegetables rather than the grains, which would be potentially reduced by these foods being consumed by livestock firstly.

## Introduction

Currently, heavy metal contamination of soil is a significant worldwide environmental problem. Agricultural soils are significantly influenced by heavy metals derived from anthropogenic activities. Nowadays, arable land contaminated by heavy metals accounts for about 20 % of all arable land in China, and the contamination levels of cadmium are higher than those of the other metals (Wei and Yang 2010; Wang et al. 2014). In a study by Niu et al. (2013), Cd had the highest pollution index (PI) of 5.28 in farmland soils across China, and Cd is a top priority heavy metal to monitor in soil for all the metals (Luo et al. 2009). Cd is one of the most hazardous heavy metals, exerting toxic effects on the kidneys, and skeletal and respiratory systems, and classified as a human carcinogen by body inhalation (IPCS 1992, 2005–2007; WHO 2010). There is a high bioaccumulation index of Cd in plants grown in soil, which causes no adverse influence on plant growth and development (Grant et al. 1998). Accordingly, Cd can readily enter the food-chain via soil-crop systems, leading to potential food safety and human health risks (Liu et al. 2006; Bernard 2008).

For the clean-up of heavy metals from soil, phytoremediation using plants, such as trees, ornamentals, and grasses, has been proposed as an environmentally friendly and cost-effective technique (Sun et al. 2009; Sarma 2011; Ji et al. 2011). However, there is a potential threat to human health in the remediation of heavy metal-contaminated farmland by crop production (Vamerali et al. 2010). Therefore, the feasibility of using crops that accumulate metals at low enough levels in their edible parts, but at high levels in their non-edible parts to remove heavy metals from agricultural soils has been discussed (Ciura et al. 2005; Murakami et al. 2009). These techniques could consider using the remediation of heavy metals from the soil, food safety, and commercial demand. Cd accumulates to different levels in different species and different cultivars within species that are called CSCs (Cd-safe cultivars) (Grant et al. 1998; Yu et al. 2006; Liu et al. 2010), as well as different distribution patterns of Cd among species in many studies (Choudhary et al. 1994; Yang et al. 1995; Grant et al. 1998). However, these studies typically focused on just the food safety, as determined by the low level of metal accumulation in the edible parts of plant, while ignoring the ability of remediation of the heavy metals from soil. Also, few studies demonstrated differences of Cd uptake depending on crop rotation (Oliver et al. 1993; Pavlíková et al. 2007). For a traditional agricultural area, intercropping and crop rotation systems have long been adopted by local farmers. Given current mild-to-moderate Cd contamination in arable soils, it is valuable and necessary to evaluate the potentially different integrated effects of these systems with accounting for remediation efficiency, distribution pattern of Cd in crops, and food safety.

A thorough understanding of the effect of production practices on Cd accumulation in crops may help in the development of management packages to reduce Cd input into the human diet. The lower reaches of the Minjiang River is one of the most important agricultural areas of the Sichuan Basin, where Wutongqiao County is the traditional agricultural and industrial base and the main ginger producing base of the Sichuan province (Liu et al. 2011). In a previous study of the heavy metal soil contamination in this area, Cd contamination was the severest comparing to the other heavy metals (Cu, Pb, Zn, Ni, and Cr), and Cd levels were 10.38 times more than the background value of Sichuan province (Du et al. 2006). Cadmium is accumulated in soils and catchments under certain environmental conditions, increasing the risk of future exposure through food. Therefore, in view of the narrow margin of safety, every effort should be made to make further reductions regarding cadmium emission into soil. We are trying to carry out the experiment of remediation of contaminated soil to build a reasonable agricultural structure for agricultural cleaner production and provide basic guarantee for the food safety of the Sichuan Basin. In this area, various typical intercropping and rotation systems with different crops have long been used. Therefore, field experiments using three typical intercropping and rotation systems in this area were conducted to: (1) investigate the different abilities of different cultivated systems for Cd removal from soil, (2) quantify the different concentrations and distribution patterns of Cd in different crops, and (3) assess the food safety by calculating the daily intake levels of Cd when consuming these crops.

## Materials and methods

### Field site and experimental design

**Fig. 1:**
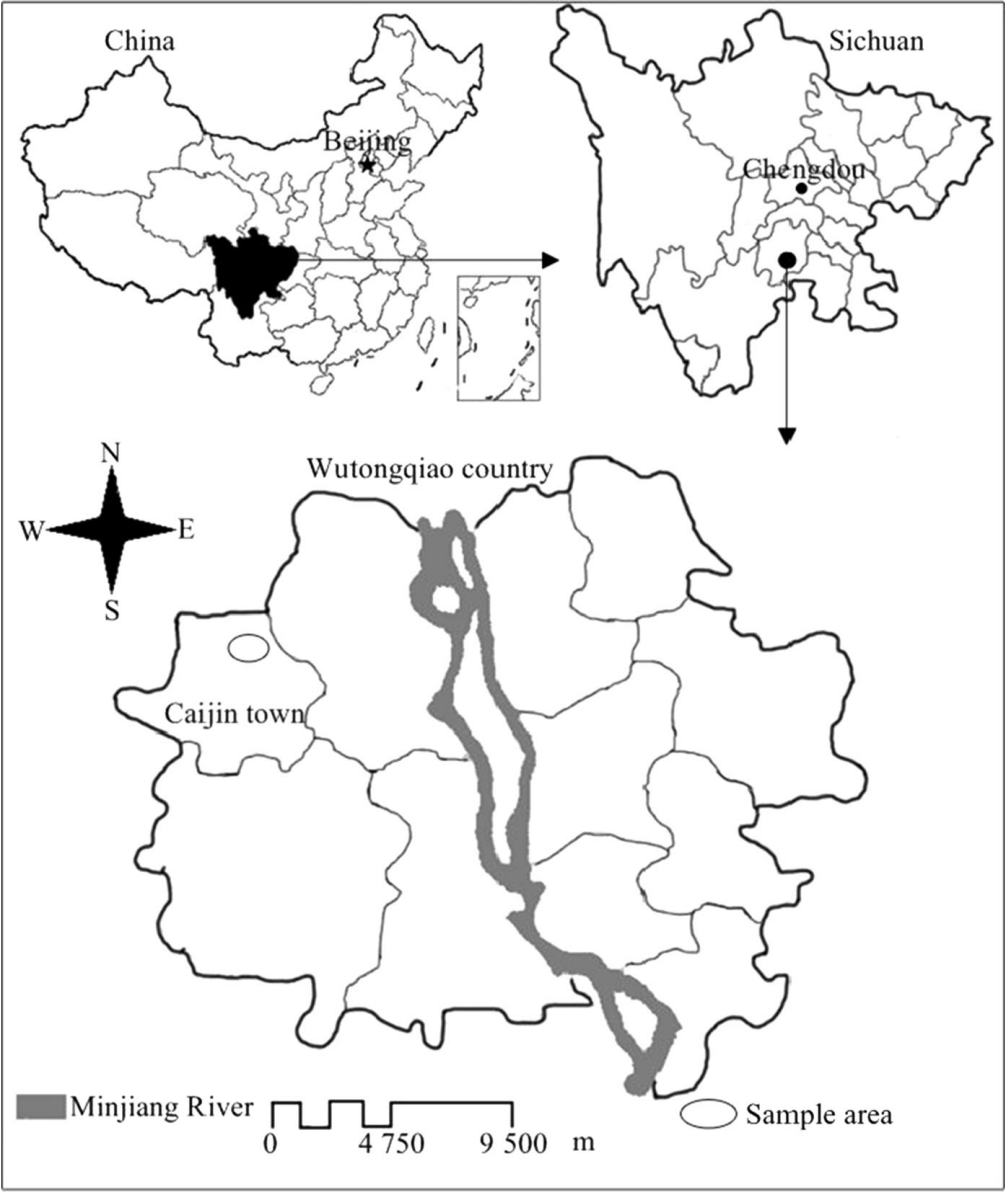
Geographical location of study area in the lower reaches of Minjiang River in south-western of China

Wutongqiao County (29°17′29″–29°31′30″ N, 103°39′45″–103°56′48″ E, a.s.l. 342–950 m) is located about 130 km southwest of Chengdu city in south-western China, belonging to the plain-hilly transitive zone with a basin-plain in the Sichuan province. The Minjiang River runs longitudinally through Wutongqiao County from north to south. It is in a subtropical zone with a warm and moist climate with average annual temperature of 17.3 °C, average annual precipitation of 1390.6 mm, and sunshine times per year of 1119.7 h. The predominant soil types include: purple soil, alluvial soil, and yellow soil (by FAO Taxonomy). The experiment fields (29°26.43′N, 103°41.67′E, a.s.l. 379 m) are located in Caijin town, a major agricultural production area of Wutongqiao Country (Fig. 1).

**Fig. 2:**
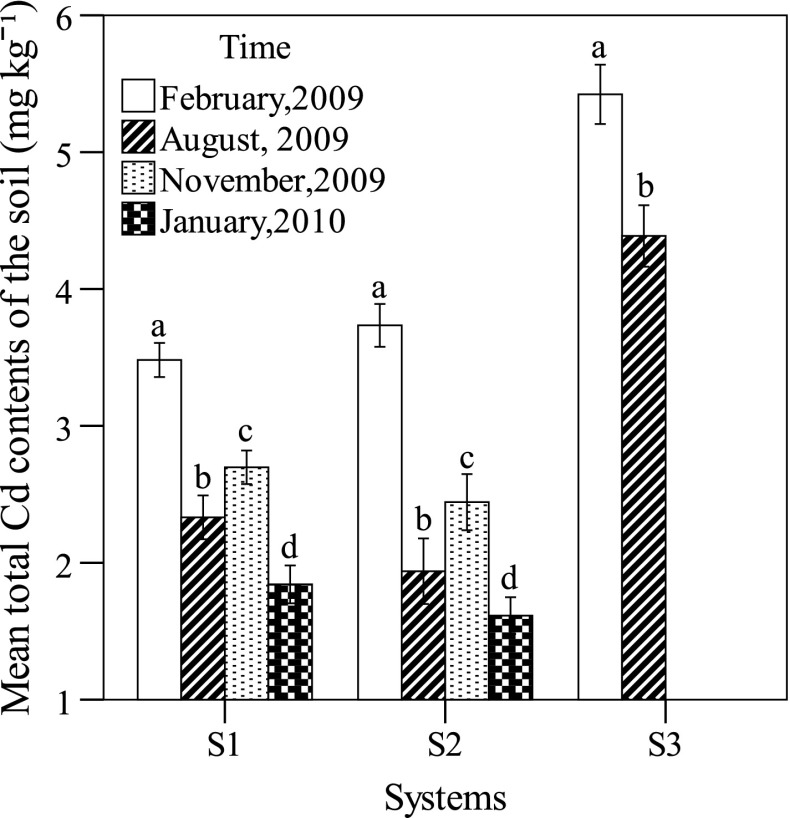
Mean total Cd levels in the soil affected by the three cropping systems. The *vertical bars* represent ± SD. *Different letters* within each system indicate significant differences between crop rotation (sampling time) at the 0.05 level according to one-way analysis of variance or *t* tests (*n* = 90). S1: maize + sweet potato—Chinese cabbage, S2: maize + ginger—stem mustard, and S3: rice

Three cropping systems with six crops that represent the major cultivated systems in past long years in this area were studied, including S1: maize (*Zea mays* L.) + sweet potato (*Ipomoea batatas* L.)—Chinese cabbage (*Brassica rapa* L. Chinensis Group.); S2: maize (*Z. mays* L.) + ginger (*Zingiber officinale* R.)—stem mustard (*Brassica juncea Coss.* var. tsatsai Mao); and S3: rice (*Oryza sativa* L.). The “ + ” means intercropping, and “–” means rotation. The conditions of the experiment fields and the physical–chemical parameters of the soils are listed in Table 1. Three cropping systems were grown from February 2009 to January 2010 under normal arable management by local farmers (Fig. 2).

**Table 1:** Soil physical–chemical characteristics (mean ± SD, dry weight) of the three cropping systems in Caijin in Wutongqiao County located in the lower reaches of the Minjiang River in the southwest of China

Systems	Plot area (m^2^)	Gradient (°)	Aspect	Agro type (by Chinese system)	Bulk density (g cm^−3^)	pH	OM (g kg^−1^)	TN (g kg^−1^)	AN (g kg^−1^)	TP (g kg^−1^)	AP (g kg^−1^)
S1	56	6.3	NE36°	Purple soil	1.26	7.81	29.12	1.38	0.104	0.78	0.022
S2	54	4.7	NE98°		1.31	7.78	25.36	1.44	0.118	0.78	0.015
S3	52	5.8	NE60°		1.48	6.11	39.66	2.44	0.166	0.36	0.001

S1: maize + sweet potato—Chinese cabbage, S2: maize + ginger—stem mustard and S3: rice

*OM* organic matter content, *TN* total nitrogen content, *AN* available nitrogen content, *TP* total phosphorus content, and *AP* available phosphorus content

(*n* = 27)

### Sampling and measurement of Cd concentration

Crop samples were collected at harvest time from fields along with accompanying soil samples (Table 2). Five sample plots (1 m^2^) were selected randomly and distinguish the different organs by calculating the water coefficient to estimate the crop biomass per square metre. Only the aboveground parts of maize and rice were collected, while the whole plants including roots were collected for the other crops. In S3, the growth time of rice was from February to August 2009 and then the field was left fallow after harvesting, so no soil or crop samples were collected from S3 after rice harvesting. Ten individuals of each crop from each system were randomly collected once at harvest time, rices, sweet potato, Chinese cabbage, ginger, stem mustard were sampled in different times, maize was sampled simultaneously in S1 and S2, so we analysed 70 crops samples in total. Nine soil samples at 0–20 cm in depth were sampled at each time, soil of S1 and S2 were sampled 4 times (9 × 2 system × 4 times), while soil of S3 was sampled 2 times (9 × 1 system × 2 times), so we analysed 90 soil samples in total.

**Table 2:** Sampling times and materials

Systems	Samples	Times and materials			
		February, 2009	August, 2009	November, 2009	January, 2010
S1	Soil	*y*	*y*	*y*	*y*
	Crop	*n*	Maize	Sweet potato	Chinese cabbage
S2	Soil	*y*	*y*	*y*	*y*
	Crop	*n*	Maize	Ginger	Stem mustard
S3	Soil	*y*	*y*	*n*	*n*
	Crop	*n*	Rice	*n*	*n*

*y*: materials were collected, and *n*: no materials were collected. S1: soil samples *n* = 36, crop samples *n* = 30; S2: soil samples *n* = 36, crop samples *n* = 30; S3: soil samples *n* = 18, crop samples *n* = 10

After air-drying at room temperature, the soil samples were put through a 2-mm nylon sieve, ground in an agate mortar, and then sieved through a 0.15-mm nylon sieve. After that, 0.30 g powder of each soil sample was put in a Poly Tetra Fluoro Ethylene (PTFE) beaker and dissolved completely by acid digestion using a mixture of HCl–HNO_3_–HF–HClO_4_ on hot plate at atmospheric pressure according to the procedure described by the national standard of China (GB/T 17141-1997) (MOE 1997).

Crop samples were separated based on organ for each crop (maize and rice: stem, leaf, and grain; sweet potato and ginger: fruit (root), stem, and leaf; Chinese cabbage: root and leaf; stem mustard: root, stem, and leaf) and carefully rinsed with tap and deionized water. The same parts of the same crops in each system were mixed. Then, the samples were oven-dried at 85 °C for 10–15 min and then at 70 °C until a constant weight was obtained. Dried crop samples were ground fully in an agate mortar and sieved through a 0.15-mm nylon sieve. After that, 1.0 g of the powder from each crop sample was placed in a flask with glass beads and then dissolved completely by acid wet digestion using a mixture of acid (HNO_3_ + HClO_4_ = 4 + 1) on hot plate at atmospheric pressure according to the procedure described by the national standard of China (GB/T 5009.15-2003) (MOH and SAC 2003).

Finally, the Cd levels of the extracts from the soil and crop samples were determined using a Thermo SOLAAR flame atomic absorption spectrometer (Model: M series 650700 v1.26, Thermo Electron Company, USA). Reagent blanks, duplicate samples, and certified reference samples were included in each batch for quality control. The range limit of duplicate samples values was 15 %, and the average recovery rate was 92 %.

### Calculations

The degree of contaminated soil was assessed by using the single pollution index (SPI) (MOA 2003), which was calculated as: SPI = the total Cd content in soil/MPL_soil_, where MPL_soil_ is the maximum permitted standard limit of Cd for soil. It implies no soil Cd contamination when SPI ≤ 1, otherwise there was soil Cd contamination.

The bioaccumulation factor (BAF) of the crop organ was calculated as BAF = the Cd content in crop organ/the total Cd content in the soil (Liu et al. 2005), and was used to evaluate the phytoextraction efficiency of the crop organ for Cd from soil. The exceeding level (EL) related to standard limit for food was calculated to estimate the extent of Cd contamination in crop edible part and was determined by: EL = (the Cd content in crop edible part−the MPL_crop_)/MPL_crop_, where MPL_crop_ is the maximum permitted limit of the Cd for the crop edible part.

It is assumed that an average adult weight was 55.9 kg, and the quantity of grain, vegetables, sweet potato, and ginger consumed 389.2, 242, 77, and 5 g per day, respectively (Zheng et al. 2007). As an estimate of the daily intake amount (DI) of Cd that based on an adult consumed from food in these cropping systems, DI was calculated as: the *measured* Cd concentration of each food × the amount of this food consumed by an adult per day (Liu et al. 2005).

### Statistical analyses

The effects of crop rotation (sampling time) on Cd concentrations in the soil and Cd concentrations among organs in each crop were determined by one-way analysis of variance. The significance level of the statistical tests was 0.05. When significant effects were found, we used Tukey’s honest significant difference. The above analyses were conducted using SPSS 16.0 (IBM SPSS Statistics Inc., Chicago, IL, USA) software package.

## Results and discussion

### Cd concentration in soils and their dynamics

Cd is generally present in the environment at low level; however, human activities have greatly increased the level in this study area. Since 1960s, chemical industry and coal mining became the leading industry in this area, and smelting and mining operations contaminate the atmosphere and aquatic environment, resulting in a gradual increase in cadmium level in soils and crops. In addition, application of municipal sewage sludge to agricultural soil can also be a significant source of cadmium. Agricultural fertilizer has been a long-term measure in the agricultural production in the lower reaches of the Minjiang River of south-western China, and there were high total and available concentrations of P in the top layers of arable soils being found (Table 1). Phosphorous fertilizer applications can lead to elevated Cd in agricultural soils. Under the background of this kind of environment, soil Cd concentration in the study area indeed was very high. The Cd concentration in the soil of S1, S2, and S3 was 3.48, 3.73, and 5.42 mg kg^−1^, respectively, at the beginning of research (Table 3). This was 4.24, 4.55, and 6.61 times the average soil Cd concentration (0.82 mg kg^−1^) of Wutongqiao County according to a previous investigation (Du et al. 2006), which was already 10.38 times more than the background Cd value for soil from Sichuan province (0.079 mg kg^−1^) (CNEMC 1990). According to the MPL_soil_ of Cd for agricultural soil set by the Chinese government (Table 3) (NEPA and ATS 1995), SPIs of Cd in soils of three systems reached the values of 5.80, 6.22, and 18.07, respectively.

**Table 3:** Total Cd concentrations (mean ± SD) in the soils for the three cropping systems, maximum permitted limit (MPL_soil_), and threshold levels (mg kg^−1^, dw) for Cd in the soils

Systems	Time	Total Cd content in soil in this study	Total Cd content in soil from Wutongqiao County^a^	Natural background levels of soil^b^	Threshold of Cd in natural background soil in China^c^	MPL_soil_ of Cd in agricultural soil in China^c^
S1	Beginning^d^	3.48 ± 0.17	0.82	0.079 (in Sichuan) 0.097 (in China)	0.2	0.6 (pH > 7.5) and 0.3 (pH < 6.5)
	End^e^	1.84 ± 0.19				
S2	Beginning^d^	3.73 ± 0.22				
	End^e^	1.61 ± 0.19				
S3	Beginning^d^	5.42 ± 0.30				
	End^f^	4.39 ± 0.31				

^a^Du et al. (2006)

^b^CNEMC (1990)

^c^NEPA and ATS (1995)

^d^February in 2009

^e^January in 2010

^f^August in 2009

Accordingly, the Cd contamination of soil in this area was severe, making it necessary to evaluate the food safety of the crops from these fields and carry out remediation for the Cd contamination. Cd concentration in plough layer extracted by crops were 15.11, 8.47, and 2.50 mg/m^2^ after 1-year rotation, with the percentage of Cd taken by crops in three system fields being S1(2.30 %) > S2 (1.16 %) > S3 (0.21 %), respectively (Table 4). Their SPIs were decreased to the values of 3.07, 2.68, and 14.63 for S1, S2, and S3, respectively. This indicated that the efficiencies of the three cropping systems for removal of Cd from the soils were remarkable and S1 might have the strongest removal ability among the three systems. Andersson and Siman (1991) and Selles et al. (1996) found that the retention or the return of residues of former crops tended to increase the Cd levels in subsequent crops than the removal of residues. In this study, we did not remove the root residues of maize when harvesting maize. Therefore, both above reasons likely contribute to the reduction of Cd levels in soils when harvesting maize, but the increase in Cd concentrations in soils when sweet potato or ginger were grown relative to the former harvest.

**Table 4:** Cd concentrations (mean ± SD) (mg kg^−1^, dw) and accumulation amount (mg/m^2^) in crops from the three cropping systems

Organs	Maize^a^	Maize^b^	Rice	Sweet potato	Ginger	Chinese cabbage	Stem mustard
Roots or fruits	–	–	–	0.89 ± 0.10a	0.16 ± 0.01a	1.09 ± 0.01a	1.06 ± 0.11a
				2.94	0.23	1.09	0.32
Stems	1.71 ± 0.05a	0.71 ± 0.27a	2.28 ± 0.15a	1.81 ± 0.20b	0.20 ± 0.01a	–	1.26 ± 0.16a
	1.82	0.76	1.57	2.98	0.10	–	0.72
Leaves	2.11 ± 0.06b	2.52 ± 0.41b	2.32 ± 0.02a	2.02 ± 0.52b	0.60 ± 0.06b	1.50 ± 0.04b	1.12 ± 0.05a
	1.82	2.17	0.71	3.70	1.63	0.38	2.23
Seeds	0.15 ± 0.003c	0.12 ± 0.02c	0.18 ± 0.01b	–	–	–	–
	0.39	0.31	0.22				
Accumulation amount (mg/m^2^)	4.03	3.24	2.50	9.61	1.96	1.47	3.27

Different letters in a column indicate significant differences at 0.05 level according to the LSD test and *t* test (*n* = 70)

^a^and ^b^ represent the maize from the S1 and S2 systems, respectively

### Distribution of Cd concentration in crops

All crops had similar distribution patterns of Cd concentration in the same crop type. For grain crops (maize and rice), the distribution pattern of Cd concentration was leaves > stem > seeds, with the Cd level in the stems or leaves being 5.92–21.00 times those in grains. Similar findings were also found in other grain crops, for instance, the Cd concentration of straw was greater than that of grain in paddy (Liu et al. 2006), wheat (Hart et al. 1998), corn (Liang et al. 2005), and oat (He and Singh 1994). For the root crops (sweet potato and ginger), the distribution pattern of Cd was leaves > stem > root, with the Cd level in stem or leaves being 1.25–3.75 times those found in roots; for leaf vegetables, the distribution pattern of Cd was leaves > stem > root, with the Cd level in the stems or leaves being only 1.06–1.38 times those in the roots (Chinese cabbage and stem mustard). High metal concentration ratios (shoot/root > 1.0) are generally associated with hyperaccumulation or as one of indicators of hyperaccumulation (Brown et al. 1994; Wu et al. 2010). Thus, these crops could have good potential to remove Cd from soils and would be suitable for remediation of mild-to-moderate Cd contamination of farmlands. The leaves of all except the stem mustard crop had the most amount of Cd accumulation, with the highest value (2.52 mg kg^−1^) measured in the maize leaves from system S2. The grain was the organ with the least amount of Cd accumulation, with the lowest value (0.12 mg kg^−1^) recorded for the maize seeds from system S2.

For almost all of the crops, the edible parts accumulated lower Cd concentrations than the inedible parts, with the exception that the edible part (stem) of stem mustard was a bit higher that the inedible parts (root and leaves; Table 4). However, the difference between the edible and inedible parts in stem mustard was not statistically significant (0.077 ≤ *P* ≤ 0.550), while the differences between the edible and inedible parts in the other crops were generally statistically significant. The Cd concentrations of the edible parts among the different crops showed the following trend: cabbage (leaves) > mustard (stems) > sweet potato (roots) > rice (seeds) > ginger (roots) > maize (seeds).

**Fig. 3:**
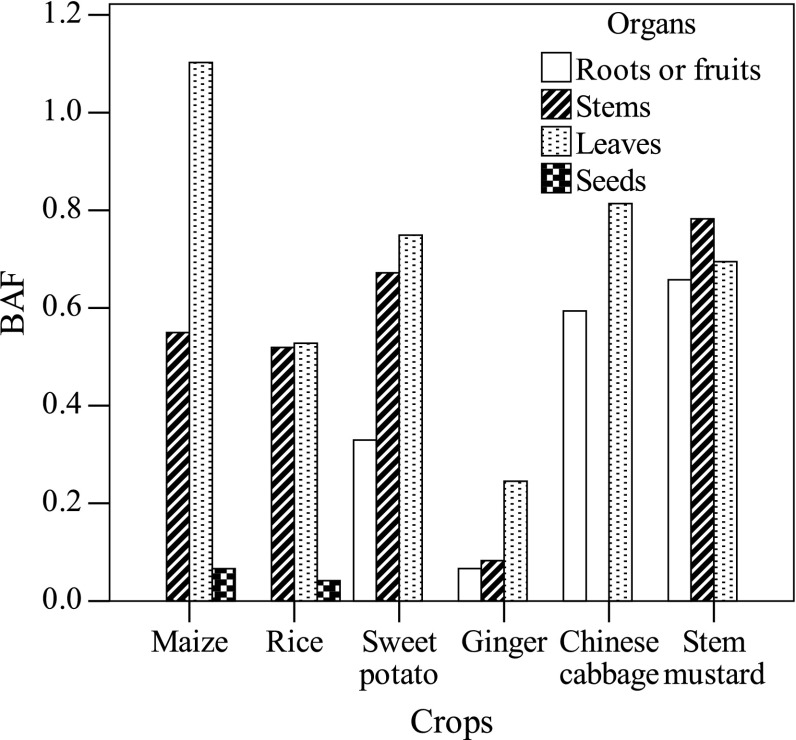
Bioaccumulation factor (BAF) of crop organs from three cropping systems. The BAFs of maize are the average values for maize organs from the S1 and S2 systems

Different BAF values for different organs would indicate their different abilities to accumulate heavy metals (Liu et al. 2005), and the BAF value of all the other organs was lower than 1.0 except maize leaves with rice seed having the lowest value (0.04; Fig. 3). The BAF values for ginger organs were in the range of 0.07–0.25, being markedly lower than those calculated for the organs of the other crops except maize seeds and rice seeds. Therefore, ginger may be an avoidance plant, defined as a plant that has the ability to suppress Cd accumulation by the plant organs (Sabreen and Sugiyama 2008). The BAF values of the stems and leaves in maize and rice were in the range of 0.52–1.10, which was much higher than those of their seeds (0.04–0.07). Thus, maize and rice (grain crops) could be used as the good crops for Cd removal from farmlands due to the considerably lower Cd accumulation in their edible organs, but the high Cd accumulation in their inedible organs. However, for leaf vegetables (Chinese cabbage and mustard), the BAF values of all their organs were somewhat high (0.59–0.81), suggesting that leaf vegetables would be affected easily by soil Cd level.

### Daily intake estimate of Cd from the three cropping systems

Brooks (1998) found that the maximum and normal levels of Cd in plants grown in the non-polluted conditions were 0.20 and 0.10 mg kg^−1^, respectively. The Cd accumulations in the edible parts of maize (seeds), rice (seeds), and ginger (roots) in this study were lower than the non-polluted conditions, while the edible parts of the other crops were much higher than this range (Table 5). Based on the daily average amount of these crops that are consumed by an adult (55.9 kg; Table 5) (Zheng et al. 2007), the total Cd intake from these three systems would reach 0.461 mg day^−1^, or 247 μg kg^−1^ body weight per month (30 d per month), which is nearly 10 times the recommended tolerable monthly intake (RTMI) of Cd (25 μg kg^−1^ body weight) established by the Joint Food and Agriculture Organization of the United Nations (FAO)/WHO Expert Committee on Food Additives (JECFA) in 2010 (WHO 2010). Additionally, based on the MPL_crop_ for foods established by the Chinese government (MOH and SAC 2005), only the edible part of rice had lower Cd leaves than its MPL_crop_. The maize and ginger edible parts had slightly higher Cd levels than their MPL_crop_, with exceeding levels (ELs) of 0.5, 0.2, and 0.6 times, respectively, while the edible parts of sweet potato, Chinese cabbage, and stem mustard had much higher Cd levels than their MPL_crop_ with ELs of 6.5–11.6 times (Table 5). Therefore, consumption of sweet potato, Chinese cabbage, and stem mustard may have a high health risk to local inhabitants.

**Table 5:** Exceeding limit (EL) and daily intake amount of Cd through consumption of foods from the three cropping systems

Type of food	Crop	Cd content in edible parts (mg kg^−1^)	MPL_crop_ of Cd content for foods in China (mg kg^−1^)^f^	EL	Hygienical standard levels for feedstuff in China (mg kg^−1^)^g^	EL′	Mean Cd content in each type of foods (mg kg^−1^)	Food intake (g day^−1^)	DI (mg day^−1^)
Cereal	Maize	0.15^a^, 0.12^b^	0.1	0.5, 0.2	0.5	n.e.	0.15	389.2^c^	0.06
	Rice	0.18	0.2	n.e.		n.e.			
Potato	Sweet potato	0.89	0.1	7.9		0.78	0.89	77^d^	0.07
Ginger	Ginger	0.16	0.1	0.6		n.e.	0.16	5^e^	0.001
Vegetables	Chinese cabbage	1.5	0.2	6.5		2	1.38	24^c^	0.33
	Stem mustard	1.26	0.1	11.6		1.52			
Total									0.46

Cereal: including maize and rice, vegetables: including Chinese cabbage and stem mustard. DI represents daily intake amount of Cd through consumption of food from the three cropping systems. EL and EL′ represent the exceeded levels related maximum permit limits (MPLcrop) for foods and feedstuff, separately, and n.e. represent none exceeded the MPLcrop

^a^and ^b^ represent the maize from the S1 and S2 systems, respectively

^c^Zheng et al. (2007)

^d^WHF (2010)

^e^HRI (2010)

^f^MOH and SAC (2005)

^g^ATS (2001)

However, in China, a majority of sweet potatoes and leaf vegetable would be cultivated not directly for food for human consumption, but rather for feeding livestock. Based on the hygienic standard level of Cd metal for feedstuff (ATS 2001), the edible parts of maize, rice, and ginger were in the safe level. However, the sweet potato and vegetable edible parts had somewhat higher Cd concentrations than the hygienic standard level, with the exceeding levels (EL′) of 0.78–2.00 (Table 5). Consequently, sweet potatoes and vegetable consumed by livestock may reduce the health risk of Cd toxicity in human.

## Conclusions

Results from this study provided valuable information not only for agricultural soil Cd contamination, but also for assessing the safety of food from crop rotation management. Although the Cd contamination of soils in the lower reaches of Minjiang River was found to be 10.38 times the background level of the Sichuan province, some sites in this area have worse Cd soil contamination, making the remediation Cd contamination from these soils and/or evaluating the safety of food from this field imperative. Three typical intercropping and rotation systems used in this study all exhibited the ability to remove Cd from soils to some degree, and the Cd reductions by crops extraction were about 0.2–2.3 % of soil levels. However, the retention of plant residues, such as the roots, after harvesting would return Cd into soils again, leading to re-contamination. Therefore, for the remediation of Cd-contaminated soils, it would be better to clear residues out of fields after harvesting. The same kind of crops had the same pattern of Cd distribution in organs as observed in grain crops (leaves > stem > seeds), root crops (leaves > stem > root), and leaf or stem vegetables (leaves or stem > root). Generally, the edible parts accumulated lower amounts of Cd than the inedible parts, suggesting that these crops, especially the grain crops, might be suitable for use as phytoaccumulators of Cd for farmlands. However, direct consumption of food made from the crops of the three systems would pose a high health risk since the monthly intake of Cd (247 μg kg^−1^ body weight) would be nearly up to 10 times higher than the PTMI (25 μg kg^−1^ body weight); however, vegetables consumed by livestock indirectly would be potentially lowered this risk.
